# Detoxification mechanisms of honey bees *(Apis mellifera)* resulting in tolerance of dietary nicotine

**DOI:** 10.1038/srep11779

**Published:** 2015-07-02

**Authors:** Esther E. du Rand, Salome Smit, Mervyn Beukes, Zeno Apostolides, Christian W.W. Pirk, Susan W. Nicolson

**Affiliations:** 1 Department of Biochemistry, University of Pretoria, Private Bag X20, Hatfield, 0028, South Africa; 2 Department of Zoology and Entomology, University of Pretoria, Private Bag X20, Hatfield, 0028, South Africa; 3 Proteomics Unit, Central Analytical Facility, Stellenbosch University, Private Bag X1, Matieland, 7602, South Africa

## Abstract

Insecticides are thought to be among the major factors contributing to current declines in bee populations. However, detoxification mechanisms in healthy, unstressed honey bees are poorly characterised. Alkaloids are naturally encountered in pollen and nectar, and we used nicotine as a model compound to identify the mechanisms involved in detoxification processes in honey bees. Nicotine and neonicotinoids have similar modes of action in insects. Our metabolomic and proteomic analyses show active detoxification of nicotine in bees, associated with increased energetic investment and also antioxidant and heat shock responses. The increased energetic investment is significant in view of the interactions of pesticides with diseases such as *Nosema* spp which cause energetic stress and possible malnutrition. Understanding how healthy honey bees process dietary toxins under unstressed conditions will help clarify how pesticides, alone or in synergy with other stress factors, lead to declines in bee vitality.

The honey bee *Apis mellifera* is an ecologically and economically important pollinator species worldwide. Recent declines in bee populations have prompted numerous studies on the factors that influence their vitality^1^,^2^. No single cause has been identified for the sometimes dramatic overwintering losses of honey bees but rather multiple interacting factors, such as pesticides, malnutrition, habitat loss, parasites and pathogens have been suggested as causing chronic sublethal stress^2^,^3^,^4^. Of all the stress factors a group of neurotoxic pesticides, the neonicotinoids, has been singled out due to its extensive use in crop protection^5^.

These nicotine-like compounds used for the protection of agricultural crops are systemic insecticides and trace residues can be found in nectar and pollen^6^, exposing bees that forage on these flowers. Most acute and chronic toxicity studies have been performed under laboratory conditions as semi-field and field studies assessing toxicity are challenging (controlled multifactor experiments at the landscape scale are very hard to conduct)^5^,^6^. As a result it is not yet clear how much neonicotinoid-containing nectar and pollen is collected and stored in bee hives^7^, or how much can be tolerated on a colony level^4^,^5^. Apart from the acute and chronic toxicity effects, sublethal effects of dietary neonicotinoids have also raised concerns. Neonicotinoids have been demonstrated to adversely affect bee immunity^8^ as well as behavioural traits, such as communication, homing and foraging^9^.

One of the principal mechanisms used by insects to escape the adverse effects of both natural and synthetic toxins, such as nicotine and the neonicotinoids, is metabolic resistance. The major enzyme superfamilies responsible for the metabolism or detoxification of toxins are the cytochrome P450 monooxygenases (P450s), glutathione transferases (GSTs) and carboxylesterases (COEs)^10^. The sequencing and annotation of the honey bee genome revealed a 50% or greater reduction in the number of genes encoding for these enzyme families relative to other insect genomes^11^. A comprehensive review of available pesticide toxicity data^12^ found that while honey bees can be sensitive to individual insecticides, in general they are no more vulnerable than other insect species. However, it has been suggested that the smaller number of detoxification genes may limit the capacity of honey bees to metabolize multiple toxins simultaneously, causing bees to be more sensitive to synergistic interactions of pesticides e.g. competitive inhibition of P450s^13^,^14^.

Several studies have demonstrated the involvement of the P450-, GST- or COE-enzyme families in pesticide and secondary metabolite detoxification in honey bees^13^,^14^,^15^,^16^,^17^,^18^. Mao *et al.* (2009) were the first to assign a function to a specific honey bee cytochrome P450 enzyme by demonstrating that CYP6AS3 is involved in the detoxification of quercetin, a flavonol present in pollen and numerous floral nectars, while CYP9Q1, CYP9Q2 and CYP9Q3 were the first specific P450s identified to contribute to pesticide (tau-fluvalinate and coumaphos) detoxification in honey bees^17^. However, even though we are able to link specific enzymes or enzyme families to honeybee detoxification processes, the overall protection mechanisms that allow these insects to tolerate the variety of potentially toxic secondary metabolites and pesticides encountered in floral nectars and pollen remain largely unknown.

Nicotine is a highly toxic alkaloid found primarily in the plant family Solanaceae, including tomato, potato, green pepper and tobacco. It is a broadly effective defence against herbivores, with a mode of action resembling that of synthetic neonicotinoids; and is used as a nonsynthetic insecticide in the form of tobacco tea in organic farming methods^19^. Nicotine mimics acetylcholine at the neuromuscular junction in mammals, causing twitching, convulsions and even death^20^,^21^. In susceptible insects, the same mode of action is observed in the ganglia of the central nervous system^21^. Only a few insect species such as *Myzus persicae* (susceptible strains LC_50_ < 30 ppm; resistant strains LC_50_ > 200 ppm), *Bemisia tabaci* (resistant strains LC_50_ = 2000–10 000 ppm) and *Manduca sexta* are known to tolerate nicotine in their diet^22^,^23^,^24^. Honey bees encounter nicotine in the nectar and pollen of *Nicotiana* spp at levels between 0.1–5 ppm in nectar and up to 23 ppm in pollen^25^,^26^,^27^. Studies investigating the effect of dietary nicotine on the survival of bees showed not only that naturally occurring concentrations of nicotine do not deter honey bees, but also that nectar nicotine at concentrations up to 50 ppm (300 μM) had no notable detrimental effects on worker survival, hatching success or larval survival^27^,^28^,^29^,^30^. In comparison to nicotine-sensitive aphids which showed 100% mortality after only six days on a diet containing 30 ppm nicotine^22^, honey bees tolerated 50 ppm nicotine in their diet during 21 day survival studies^29^ (honey bee LC_50_ = 2000 ppm^26^).

In the insect species that have adapted to nicotine-producing plants, such as *M. persicae, B. tabaci* and *M. sexta*, nicotine tolerance is linked to cytochrome P450-mediated detoxification^22^,^23^,^31^. It is widely assumed that metabolic detoxification mechanisms in insects are energetically expensive^32^,^33^, however, the absence of evidence for costs has also been reported^34^,^35^. Previously, we demonstrated that dietary nicotine had no significant adverse effects on lipid and protein reserves in honey bee larvae^28^, suggesting negligible energetic or metabolic costs associated with the observed nicotine tolerance.

To date, studies investigating the mechanisms involved in detoxification of pesticides and secondary metabolites in honey bees have utilized *in vitro* metabolism assays (enzyme assays) or toxicity bioassays in the presence or absence of known inducers or inhibitors of specific enzyme families involved in detoxification^14^,^15^,^16^,^17^,^36^. Systems biology approaches include genome-wide transcriptome analysis of specific tissues such as the midgut or the abdomen^36^,^37^,^38^ or transcriptomic analysis of selected detoxification, immune and stress response genes^14^,^17^,^39^ after pesticide or xenobiotic exposure. However, mRNA expression profiles do not always correlate with protein concentrations and metabolic state. Therefore, in this study we employed an integrated proteomic and metabolomic approach to attain a global overview of the response of honey bees to a three day nicotine exposure. The main objective was to identify protein networks and metabolic pathways involved in the detoxification of nicotine using mass spectrometry-based comparative proteomic and metabolomic analysis. Our study revealed a complex response involving detoxification, oxidative and general stress response pathways concurrent with an increase in the insect’s energy metabolism.

## Results

Global metabolite profiles were determined in newly emerged worker bees from six colonies (n = 6) and compared between dietary treatment groups. A total of 414 metabolites were identified using GC- and LC-MS analysis, but the levels of only eight were significantly altered (Table 1). As expected, nicotine was detected only in nicotine exposed samples, along with two known metabolites of nicotine^24^,^40^, cotinine and cotinine-N-oxide. Compared to the control samples, the levels of β-hydroxyisovalerate (valine, leucine and isoleucine metabolism), 1-palmitoylglycerophophoinositol (20:0) (lysolipid metabolism) and 4-hydroxyhippurate (benzoate metabolism) were substantially higher in the nicotine exposed samples. 4-Hydroxyhippurate is a metabolite characteristic of gut microbial fermentation of polyphenols in humans^41^. Lower levels of fumarate, from the tricarboxylic acid (TCA) cycle, and cytidine 5’-diphosphocholine (glycerolipid metabolism) were also observed.

To investigate the proteomic response of honey bees following a three day nicotine exposure, we performed label free, mass spectrometry-based proteomic analysis. Global protein profiles were determined in newly emerged worker bees from three colonies (n = 3) and compared between dietary treatment groups. A total of 1470 proteins was identified with 96 substantially up-regulated and 59 down-regulated in the nicotine exposed samples (Entire lists of the differentially expressed proteins, including accession numbers, fold change and predicted functions can be found in Supplementary Tables 1 and 2).

Of the proteins that were up-regulated following nicotine exposure, the two largest groups had functions relating to energy metabolism and carbohydrate metabolism, with 15 and 16 proteins of the total 96 up-regulated proteins falling into these two groups (Table 2). Ten proteins were involved in detoxification, heat shock and antioxidant responses with four being involved in lipid metabolism, three in amino acid metabolism (specifically branched chain amino acid metabolism), and two in glutathione metabolism. The rest of the up-regulated profile consisted of proteins involved in: translation (seven); protein processing, folding, transport and modification (six); signal transduction (five); cellular processes such as transport, catabolism and cell communication (three); nucleotide metabolism (three); transcription (one) and the olfactory system (one). Twenty of the up-regulated proteins had unknown functions.

Of the 59 proteins that were down-regulated, six are involved in signal transduction, five in protein processing, folding, transport and modification, while four are involved in energy metabolism, four in muscle development and contraction; and another four are involved in cellular processes such as transport, catabolism and cell communication. The remaining down-regulated proteins had functions related to: translation (four); the cytoskeleton (three); carbohydrate metabolism (two); lipid metabolism (one); cofactor and vitamin metabolism (one); and nucleosome assembly (one). Twenty four proteins had unknown functions (Table 2).

**Figure 1:**
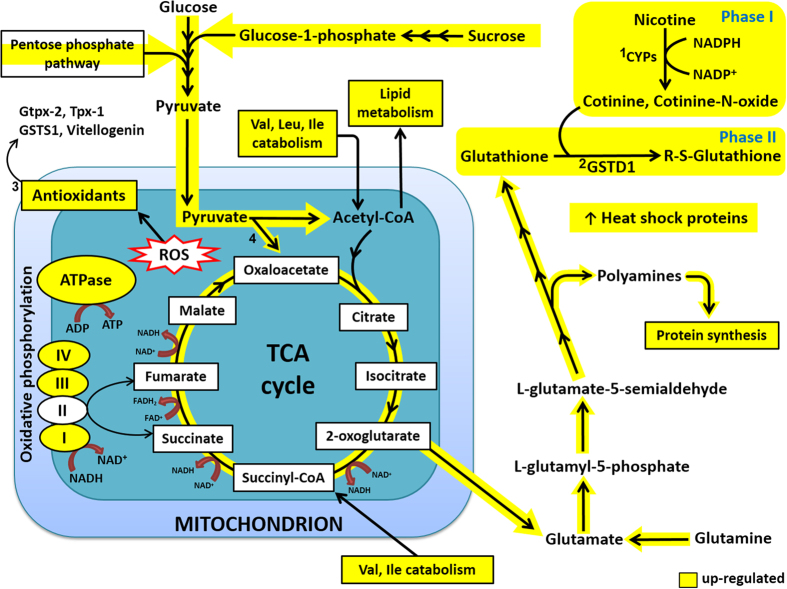
Scheme summarizing the proposed mechanisms underlying the response of honey bees to nicotine exposure. Nicotine is oxidised to cotinine and cotinine N-oxide (phase I detoxification) (1), followed by phase II conjugation catalysed by GSTD1 (2). The up-regulation of enzymes involved in glycolysis, the TCA cycle and oxidative phosphorylation suggests increased energy metabolism to support detoxification and the stress response. This leads to increased ROS production which induces enhanced expression of enzymatic and non-enzymatic antioxidants: GSTS1, phospholipid-hydroperoxide glutathione peroxidase (Gtpx-2), peroxiredoxin (Tpx-1) and vitellogenin (3). The up-regulation of the GSTs and Gtpx leads to the up-regulation of glutathione production, which is essential for the functioning of these enzymes. Increased flux through the TCA cycle provides precursors to support the increased synthesis of glutathione. The intermediates of the TCA cycle are replenished by the anaplerotic reaction pyruvate → oxaloacetate catalysed by pyruvate carboxylase (4). Increased catabolism of the branched chain amino acids meets the increased demand for acetyl-CoA due to the increased flux though the TCA cycle and the observed increased lipid metabolism. Heat shock proteins are up-regulated as part of the cellular stress response which promotes stress tolerance. ROS: reactive oxygen species; GSTD1: glutathione S-transferase delta isoform 1; GSTS1: glutathione S-transferase sigma isoform 1.

To gain further insight into the molecular mechanisms activated or repressed after nicotine exposure, we mapped the metabolites with altered levels and the differentially expressed proteins to pathways from the Kyoto Encyclopedia of Genes and Genomes (KEGG) database^42^. The constructed pathway diagram in Fig. 1 illustrates the response of honey bees to nicotine exposure for three days. Our analysis indicated an increase in phase I and phase II detoxification processes accompanied by an increase in energy metabolism (oxidative phosphorylation, glycolysis, TCA cycle, branched amino acid catabolism, and the pentose phosphate pathway), glutathione anabolism, lipid metabolism and protein synthesis, as well as increased abundance of proteins that function as part of the cellular heat shock and oxidative stress responses.

### Evidence of both phase I and II detoxification

Nicotine exposed bees exhibited detectable levels of nicotine and two of its known metabolites, cotinine and cotinine N-oxide. Considering that cotinine and cotinine N-oxide have virtually no toxicity to insects^43^, the conversion of nicotine to these metabolites suggests that honey bees actively detoxify nicotine. Snyder *et al.*^31^ demonstrated that the tobacco specialist caterpillar, *M. sexta*, metabolizes nicotine to cotinine and cotinine N-oxide via a P450-mediated pathway, supporting the notion that the C-oxidation of nicotine by honey bees constitutes true detoxification. In the nicotine-resistant peach-potato aphid (*M. persicae*) overexpression of a cytochrome P450 (CYP6CY3) allows these insects to efficiently detoxify nicotine to cotinine and aminoketone^22^, resembling a minor P450-mediated pathway in nicotine metabolism in humans. In humans, the C-oxidation of nicotine to cotinine is the major pathway of nicotine metabolism and cotinine is used as a biomarker for nicotine exposure^40^. Cytochrome P450s (mainly CYP2A6 and CYP2B6) mediate the formation of the intermediate nicotine Δ1’(5’)-iminium ion, which is subsequently oxidised to cotinine by aldehyde oxidase^40^. Cotinine N-oxide is a minor metabolite produced when cotinine is further oxidised by a cytochrome P450-mediated reaction^40^. Taken together, these observations suggest that an analogous cytochrome P450-mediated pathway is present in honey bees to facilitate nicotine catabolism.

In total twelve P450s were identified (Supplementary Table 3 lists all the P450s identified), but none were differently expressed compared to the controls, suggesting that these enzymes are constitutively expressed. This is not entirely unexpected: previous studies found that the inducibility of honey bee P450s does not necessarily correspond precisely with their metabolic activities as is often assumed from mammalian and other insect studies^14^,^16^,^17^. For example, CYP6AS3 is not inducible by its substrate, quercetin or by phenobarbital, a reliable inducer of P450 activity in many insects^14^.

Most of the identified P450s are members of the CYP6 and -9 families that are generally associated with environmental response and detoxification functions in other insects^10^. Honey bees seem to follow suit. The three enzymes identified from the CYP9Q subfamily are associated with pesticide detoxification, with CYP9Q1 and CYP9Q3 also able to efficiently metabolize the nectar and pollen flavonol, quercetin^17^. Consistent with our observations, CYP9Q2 and CYP9Q3 are known to be constitutively expressed at low levels in bees^17^. Furthermore, four enzymes from the CYP6AS subfamily (known to metabolize quercetin) were identified of which CYP6AS10 and CYP6AS15 were previously shown to be consistently expressed at high levels in bees^14^. In addition, nicotine resistance in both *B. tabaci* and *M. persicae* is associated with members of the CYP6 family^22^,^23^. It seems reasonable to propose that one or more members of the CYP6 and CYP9 families that are constitutively expressed are responsible for the phase I detoxification products of nicotine observed in the metabolomic analysis.

Two phase II enzymes, glutathione S-transferase Delta isoform 1 (GSTD1; GB50265) and glutathione S-transferase Sigma isoform 1 (GSTS1; GB48905) were also significantly up-regulated. The GST family is a multifunctional enzyme superfamily that plays important roles in the detoxification of secondary metabolites and insecticides, as well as in protection against oxidative stress^10^,^44^. In general, GSTs act by conjugating the thiol group from glutathione (GSH) to reactive oxygen species (ROS) and other toxic compounds that possess an electrophilic centre. By this mechanism, they can eliminate substrates from a cell by rendering them more water soluble and targeting them to specific GSH multidrug exporters. Insect GSTs are classified into at least six classes: delta, epsilon, omega, sigma, theta and zeta, of which the delta and epsilon classes are unique to insects. Although many GST cDNAs have been sequenced from different insect species, little is known about functional specificities of GSTs in different classes. Delta GSTs have been implicated in insecticide resistance in several insects including *B. tabaci*^44^. The putative function of *A. mellifera* GSTD1 is direct detoxification of xenobiotics (KEGG)^42^. This enzyme is widely distributed in *A. mellifera* tissues^45^ supporting a function in general detoxification. We hypothesize that GSTD1 is involved in the phase II detoxification of nicotine.

The function of GSTS1 is as yet unidentified. The discovery that GSTs from the sigma class have a high affinity for lipid peroxidation products along with the localization of these proteins in metabolically active tissues in flies^46^ led to the postulation that these enzymes may play a role in protection against oxidative stress. GSTs from this class have been associated with pesticide resistance through both direct metabolism^47^ and by protecting against lipid peroxidation damage^48^. In the sister species of *A. mellifera, Apis cerana*, GSTS1 is postulated to play a role in detoxification mechanisms and protection against oxidative stress^49^. In *A. mellifera,* sigma GSTs have been associated with the protection of the spermatozoa and the venom gland tissues against oxidative damage^50^,^51^. This together with the presented results suggests a similar role for GSTS1 of protecting tissues against oxidative damage caused by the enhanced production of ROS associated with the observed increase in energy metabolism.

### Altered energy metabolism due to increased energy demand

Resistance to plant and synthetic toxins is associated with fitness costs and in insects metabolic resistance often involves an energy cost^23^,^35^. Fifteen proteins involved in complexes I, III, IV and ATPase of the oxidative phosphorylation (OXPHOS) pathway were strongly up-regulated in nicotine exposed bees. In eukaryotes, OXPHOS is the essential part of the metabolic pathway to supply energy. The up-regulation of the OXPHOS pathway is indicative of an increased energy demand, most likely due to the activation of detoxification mechanisms and other defence mechanisms. In other insects, including *Sitophilus zeamais*^32^ and *B. tabaci*^52^, resistance to plant toxins and pesticides has also been associated with the increased expression of genes and proteins involved with OXPHOS and energy metabolism.

The observed increased expression of several proteins involved in carbohydrate catabolism (total 16), including the TCA cycle adds support to the idea of increased energy requirements during nicotine exposure and detoxification. Glycolysis seems to be up-regulated, with enolase (GB54753) and pyruvate dehydrogenase (GB53566, GB55496; pyruvate → acetyl-CoA) up-regulated 2- and 3-fold, respectively. Enzymes involved in sucrose uptake were also up-regulated. Phosphoglucomutase (GB54661), a protein involved in the processing of disaccharides to glucose, showed higher levels, as well as aldose/aldehyde reductase (GB18109) and sorbitol dehydrogenase (GB42385). In addition, the up-regulation of glycogen phosphorylase (GB42835) is indicative of increased glycogenolysis for energy production through the glycolytic pathway (Fig 1). The observation that several proteins involved in the pentose phosphate pathway (GB55537; GB54661) and pentose interconversions (GB51283; GB50272) that feed into the glycolytic pathway were also up-regulated supports the notion of increased glycolytic flux and energy demand during nicotine exposure and detoxification.

The observed increased expression of the anaplerotic enzyme, pyruvate carboxylase (GB4028; pyruvate → oxaloacetate) leads to higher levels of oxaloacetate, resulting in an increased flux through the TCA cycle. Increased flux through the TCA cycle is reflected by the up-regulation of the three rate limiting enzymes, citrate synthase (GB52073), isocitrate dehydrogenase (GB45258) and 2-oxogluterate dehydrogenase (GB44430). The reaction catalysed by both isocitrate dehydrogenase and 2-oxogluterate dehydrogenase produces NADH which feeds into the OXPHOS pathway to produce ATP.

Besides producing NADH which drives the OXPHOS pathway, the TCA cycle also produces intermediates for biosynthetic pathways. The enzyme responsible for the conversion of the TCA cycle intermediate α-ketoglutarate to glutamate, glutamate synthase (GB147100), was up-regulated 2-fold. This enzyme is also responsible for the conversion of glutamine to glutamate. Glutamate is required for the biosynthesis of glutathione which is required for the functioning of GSTs. The ultimate enzyme in the biosynthetic pathway of glutathione, glutathione synthase (GB147100) was up-regulated (2-fold) suggesting increased synthesis of glutathione. Increased synthesis of glutathione reflects the increased demand for glutathione induced by the up-regulation of GSTD1 and GSTS1 during the detoxification of nicotine. Draining of α-ketoglutarate to glutamate for glutathione synthesis would lead to the depletion of oxaloacetate. Replenishing oxaloacetate requires the up-regulation of anaplerotic or “re-filling” reactions^53^ as demonstrated by the observed up-regulation of pyruvate carboxylase (GB4028; pyruvate → oxaloacetate).

Apart from glutathione synthesis, glutamate is also used in the synthesis of polyamines for DNA/RNA and protein biosynthesis. Proteins involved in protein synthesis were substantially up-regulated in nicotine exposed bees, indicating the induction of proteins directly or indirectly involved in the mechanisms underlying nicotine tolerance (see Table 2 and Supplementary Table 1).

Interestingly, proteins involved in lipid metabolism were also up-regulated (see Table 2 and Supplementary Table 1). An increase in lipid metabolism was confirmed by the variation in the abundances of metabolites such as cytidine 5’-diphosphocholine and 1-palmitoylglycerophosphoinositol. However, the role of increased lipid metabolism in nicotine tolerance requires further investigation.

### Branched chain amino acid catabolism

Nicotine exposed bees exhibited increased expression of 3-ketoacyl CoA thiolase (GB50970), 3-hydroxyacyl-CoA-dehydrogenase (GB13680, GB55232) and retinal dehydrogenase 1-like (GB51283). These enzymes are indicative of increased catabolism of the branched chain amino acids leucine, isoleucine and valine. This observation was confirmed by the metabolomic profiling which revealed increased levels of β-hydroxyisovalerate, an intermediate in the branched amino acid catabolic pathway. Branched amino acids can contribute to energy metabolism as energy source, but the breakdown of these amino acids also replenishes or expands the pool of TCA cycle intermediates that are used as precursors in biosynthetic pathways such as the synthesis of glutathione^54^.

### Enhanced ROS production caused by the detoxification process and increased energy metabolism induces oxidative and heat shock stress responses

ROS are generated as by-products of energy metabolism and in insects increased ROS production has also been associated with P450-mediated detoxification processes^55^. Antioxidant enzymes play key roles in regulating the intracellular ROS balance to prevent ROS-mediated damage and are produced by cells in response to oxidative stress^56^. Besides the GSTS1 mentioned earlier, other enzymes associated with oxidative stress resistance in insects were also up-regulated in the nicotine exposed workers. We observed a 3-fold increase in phospholipid-hydroperoxide glutathione peroxidase (Gtpx-2; GB48634) levels and a 4-fold increase in peroxiredoxin or thioredoxin peroxidase (Tpx-1; GB40232) levels. Both these enzymes are part of the primary antioxidant response and act directly on ROS molecules^56^.

Non-enzymatic antioxidants are also involved in the oxidative stress response in insects. In nicotine exposed bees, the levels of vitellogenin (GB49544) were three times higher than those found in the controls. In insects in general, vitellogenin functions as an egg yolk precursor protein that is taken up by oocytes. However, in honey bees, vitellogenin has several regulatory roles in non-reproductive functions such as hormone signalling, immunity, stress resistance, lifespan and behaviour^57^,^58^. Vitellogenin actively protects worker bees from oxidative stress due to its ROS scavenging properties^59^,^60^. It is likely that the increased level of vitellogenin in nicotine exposed bees is part of the oxidative stress response in worker bees.

Apart from antioxidant activity, other components of the cellular stress response were also up-regulated. The levels of heat shock protein (Hsp) 90 (GB40976) increased 12-fold in nicotine exposed bees and a member of the heat shock protein 70 family (heat shock protein cognate 5 or Hsc 70-5; GB42297) and a small heat shock protein (GB47475) both increased 2-fold. In the presence of abiotic and biotic stressors Hsps are up-regulated and are thought to participate in stress tolerance and promote cell survival mainly through refolding proteins and preventing their denaturation. In arthropods, Hsp90 proteins have been shown to be involved in tolerance and resistance to pesticides^61^,^62^. In *A. mellifera* specifically, increased expression of both Hsp 70 and 90 has been associated with pesticide exposure^63^.

### The role of the proteins down-regulated in nicotine exposed honey bees

A total of 59 proteins were down-regulated in nicotine exposed honey bees (Supplementary Table 2 lists all the down-regulated proteins with functions and fold change). Several are involved in signalling pathways, specifically calcium signalling pathways. One of the many physiological functions in which calcium signalling plays a key role is muscle contraction. The down-regulation of these proteins along with the up to 5-fold down-regulation of four other proteins involved in muscle contraction and development suggests a possible role in counteracting nicotine toxicity. In insects, nicotine causes hyperactivity at low doses and reduced activity and paralysis at higher doses^21^. These effects are mediated by excitatory direct binding to nicotinic acetylcholine receptors and increased dopaminergic activity. However, the purpose of down-regulating these proteins in nicotine tolerance requires further investigation.

## Discussion

Based on the data presented and information on gene expression implicated in insect resistance to pesticides and natural xenobiotics, we propose the following detoxification mechanism in honey bees as depicted in Fig. 1. Consumed nicotine is oxidised to less toxic metabolites, cotinine and cotinine N-oxide, by phase I detoxification enzymes, most likely constitutively expressed CYP6 or CYP9 enzymes; followed by phase II conjugation with glutathione catalysed by GSTD1. The up-regulation of enzymes involved in ATP synthesis, sugar catabolism, glycolysis and the TCA cycle suggests increased energy production to support or drive the detoxification processes. Increased energy production leads to increased ROS production which induces enhanced expression of enzymatic and non-enzymatic antioxidants, specifically GSTS1, Gtpx-2, Txp-1 and vitellogenin to protect against oxidative damage. Other elements of the cellular stress response, specifically Hsp 90 (which increased 12-fold), are also substantially up-regulated to promote stress tolerance. The up-regulation of GSTs and Gtpx-2 lead to the concurrent up-regulation of anabolism of glutathione, the tripeptide substrate essential for the functioning of these enzymes. Increased flux through the TCA cycle provides precursors to support the increased synthesis of glutathione.

It is worth noting that the response mechanism mediating nicotine tolerance described here for newly emerged hive bees may differ from the response mechanism in older foragers and in larvae, due to age specific protein expression patterns. Compared to hive bees, the abundance of proteins involved in energy metabolism is higher in foragers, while ROS resistance and the total detoxification capacity related to GSTs and cytochrome P450s are lower^64^,^65^. This might be due to the division of labour and the resulting skewed toxic burden. Foragers may be exposed to toxins during flight and foraging, but they do not consume large quantities of the collected contaminated pollen and nectar. Foragers consume negligible amounts of pollen but are fed protein rich jelly by hive bees before flights^66^,^67^, while younger hive bees may be exposed to toxins during the processing of incoming contaminated pollen and nectar to bee bread, honey and jelly. Alternatively, it has been suggested that that protection mechanisms in foragers may be limited to proteins indispensable for their foraging performance^64^.

This study begins to illuminate the molecular mechanisms underlying the ability of honey bees to tolerate the dietary toxin nicotine and provides fundamental insight into the detoxification processes of honey bees. Our results demonstrate that honey bees actively detoxify nicotine to cotinine and cotinine N-oxide, forming the basis of the previously reported tolerance of nicotine in honey bees; and that these detoxification processes are associated with an increase in energetic investment. The increased energetic investment is significant in view of the interactions of pesticides with possible malnutrition^37^ and with diseases such as *Nosema* spp. that lead to energetic stress^68^. Understanding how healthy honey bees process toxins under normal conditions will improve our understanding of how pesticides, on their own or in synergy with other stress factors, lead to a decline in bee vitality.

## Methods

### Sample collection and caged bees

Frames with capped worker brood were removed from *Apis mellifera scutellata* colonies maintained at the University of Pretoria apiary during summer (January – March 2013). The frames were placed in an incubator and newly emerged workers were collected from the frames within 24 h of their emergence and placed in hoarding cages (125 workers per cage). These cages were made of polycarbonate (11 × 8.5 × 7 cm) with wire mesh bottoms for ventilation. Each contained a piece of comb (5 × 5 cm). Cages were kept in an incubator (Humidity chamber HCP108, Memmert GmbH & Co. KG; Bavaria, Germany) at 34 ± 1 °C and 45% relative humidity in darkness, simulating conditions within the hive. Plastic feeding vials (10 ml) with cut feeding holes (1 × 0.5 cm) were inserted horizontally into the cages, one with water and one with experimental diet, provided fresh daily. Control groups received a standard diet consisting of 0.63 M sucrose (Merck, Darmstadt, Germany) while experimental groups received a 0.63 M sucrose diet containing 300 μM (50 ppm) nicotine (Sigma-Aldrich, Louis, MO, USA). Bees were randomly assigned to cages and cages were randomly assigned to the experimental or control groups. The dose of 300 μM nicotine was selected as the highest concentration of nicotine fed to caged bees with no significant adverse effects^29^ and it is also well below the LC_50_ of nicotine for honey bees of 2000 ppm (concentration of nicotine that caused the death of 50% of bees)^26^. Bees were fed the control and experimental diets for 72 h, then killed by freezing and stored at −80 °C until further analysis. The estimated total body load was 3 μg nicotine per bee over 72 h.

### Metabolomic profiling

Metabolomic profiling analysis was carried out in collaboration with Metabolon Inc. (Durham, NC, USA) as described by Ref. 69. Metabolite profiles were determined in three control and three treatment cages from each of six *A.m. scutellata* colonies (n = 6). Each sample contained 100 homogenised bees.

### Sample preparation

One hundred bees were lyophilised and ground with a pestle and mortar. Using a MicroLab STAR^®^ liquid handler (Hamilton, Salt Lake City, UT, USA), protein was precipitated from the samples using a series of aqueous extractions optimised for maximum recovery of small molecules (Metabolon Inc., Durham, NC, USA). The resulting extract was split into equal aliquots for liquid chromatography (LC) and gas chromatography (GC) analysis, respectively. The sample aliquots were placed briefly on a TurboVap^®^ (Zymark Corp., Hopkinton, MA, USA) to remove any residual organic solvent and subsequently frozen and dried under vacuum. The LC sample aliquots were reconstituted in either 0.1% formic acid or 6.5 mM ammonium bicarbonate (pH 8). The GC sample aliquots were derivatised using bis-(trimethylsilyl)-trifluoroacetamide (BSTFA) at 60 °C for 1 h. All samples were spiked with injection standards at fixed concentrations.

### Mass spectrometry

Non-targeted metabolite profiling was performed using three independent platforms^69^. Ultrahigh performance liquid chromatography/tandem mass spectrometry (UPLC/MS/MS^2^) optimised for acidic species, UPLC/MS/MS^2^ optimised for basic species, and gas chromatography/mass spectrometry (GC/MS) were performed. The LC/MS portion of the platform was based on a Waters ACQUITY UPLC and a Thermo-Finnigan LTQ mass spectrometer, which consisted of an electrospray ionisation source and linear ion-trap mass analyser. Sample aliquots reconstituted in acidic conditions were gradient eluted using water and methanol containing 0.1% formic acid; the basic sample aliquots were eluted with water and methanol containing 6.5 mM ammonium bicarbonate. The MS analysis alternated between MS and data-dependent MS^2^ scans using dynamic exclusion. For GC/MS analysis a 5% phenyl column and a temperature ramp of 40 to 300 °C over a 16 min period was used. Samples were analysed on a Thermo-Finnigan Trace DSQ fast-scanning single-quadrupole mass spectrometer using electron impact ionisation.

### Data analysis

Metabolites were identified by comparing the ion features in the samples to the entries of purified standards in a metabolomic reference library^69^ that includes retention time, molecular weight (*m/z*), preferred adducts, in-source fragments as well as associated mass spectra. The KEGG server^42^ was used to assign identified metabolites to specific metabolic networks in order to identify any overrepresented pathways. Missing values for a given metabolite were assigned the observed minimum value (minimum value imputation). Raw area counts for each compound were re-scaled by dividing each sample value by the median value for the specific metabolite in order to visualise the data more conveniently. R (http://cran.r-project.org) was used for the statistical analysis of the data. A log transformation was applied to the observed relative abundances and the fold change was calculated for each metabolite identified as the mean ratio of the control and treatment groups. Welch’s two-sample Student t-Tests (two-sided; alpha level set to 0.05) were used to determine whether or not each metabolite significantly increased or decreased in abundance. The false discovery rate (FDR) was calculated to correct for multiple Welch’s two-sample Student t-Test comparisons for the hundreds of compounds detected. The FDR for a given set of metabolites is estimated by the Q-value^70^. A Q-value smaller than 0.01 was used as an indication of high confidence in a result^70^. Other lines of evidence were also taken into consideration when the Q-value exceeded 0.01, such as if the metabolite shares a common pathway with a highly significant compound, or if the metabolite is in a similar biochemical functional family with other significant compounds.

### Proteomic profiling

Protein profiles were determined in three control and three treatment cages from each of three *A.m. scutellata* colonies (n = 3). Each sample contained 50 homogenised bees.

### Sample Preparation

Fifty bees were homogenised in 50 ml of lysis buffer using an Ultra-Turrax® homogeniser (IKA®-Werke GmbH & Co. KG, Staufen, Germany). The lysis buffer consisted of 7 M urea, 2 M thiourea, 4% CHAPS, 1% DTT, 40 mM Tris and Complete Protease Inhibitor tablets (Roche Diagnostics, Mannheim, Germany). After homogenisation, the samples were sonicated on ice using a Sonifier® Cell Disrupter B-30 fitted with a microtip (Branson Ultrasonics Corporation, Danbury, CT, USA) in 4 × 15 s pulses with 10 s cooling in between (settings: pulsed; Output control 3; % Duty cycle 60). Subsequently, the samples were centrifuged at 14 000 x *g* for 10 min. Trichloroacetic acid was added to the collected supernatants to a final concentration of 10%, followed by incubation on ice for 30 min to ensure the precipitation of proteins and desalting. The precipitated proteins were collected by centrifugation at 14 000 x *g* for 10 min. The collected pellets were washed three times with cold acetone. The washed pellets were dried and solubilised in 7 M urea, 2 M thiourea and 100 mM Tris (pH 8) before being stored at −80 °C. Protein concentrations were determined using the Coomassie Plus™ (Bradford) Assay Kit (Pierce, Rockford, IL, USA).

### Electrophoresis

Extracted proteins were resolved on precast 10-well 12% Mini-Protean TGX Stain Free gels (Bio-Rad, Hercules, CA) according to the manufacturer’s instructions (55 μg protein was loaded per lane). Coomassie® Brilliant Blue G250 (Merck, Darmstadt, Germany) was used to visualise the protein bands. After visualisation, each sample lane was cut into four pieces.

### In gel trypsin digestion

Gel pieces were cut into smaller cubes and washed twice with water before being washed with 50% acetonitrile. The washed gel pieces were incubated in 50 mM ammonium bicarbonate for 10 min before being incubated in 100% acetonitrile until the pieces turned white after which they were dried *in vacuo*. Proteins were reduced for 1 h at 57 °C using 10 mM DTT after which the gel pieces were washed with 50 mM ammonium bicarbonate followed by 50% acetonitrile. The proteins were alkylated with 55 mM iodoacetamide for 1 h in the dark, after which the gel pieces were washed with ammonium bicarbonate for 10 min followed by 50% acetonitrile for 20 min, before being dried *in vacuo.* The gel pieces were digested overnight at 37 °C with 100 μl of a 10 ng/μl trypsin solution. The resulting peptides were extracted with 70% acetonitrile containing 0.1% trifluoroacetic acid for 30 min (twice) followed by a 30 min extraction step using 100% acetonitrile before being dried. The dried peptides were dissolved in 5% formic acid and cleaned using Stage Tips (Thermo Scientific, IL, USA) according to the instructions. The peptides were dried and stored at −20 °C. Dried peptides were redissolved in 5% acetonitrile in 0.1% formic acid for nano-LC analysis.

### Mass spectrometry

All experiments were performed on a Thermo Scientific EASY-nLC II connected to a LTQ Orbitrap Velos mass spectrometer (Thermo Scientific, Bremen, Germany) equipped with a nano-electrospray source. LC separations were carried on an EASY-Column (2 cm, ID 100 μm, 5 μm, C18) pre-column followed by a X-Bridge BEH130 NanoEase column (15 cm, ID 75 μm, 3.5 μm, C18). Mobile phase A consisted of 0.1 % (v/v) formic acid and mobile phase B consisted of 90% (v/v) acetonitrile and 0.1% (v/v) formic acid. The gradient used was: 0–5 min, 5–17% B; 5–95 min, 17–25% B; 95–105 min, 25–60% B; 105–110 min, 60–80% B; 110–120 min, 80% B. The flow rate was set at 300 nl/min and the injection volume was 10 μl. The mass spectrometer was operated in data-dependent mode to automatically switch between Orbitrap-MS and LTQ-MS/MS acquisition. Data were acquired using the Xcaliber software package. The precursor ion scan MS spectra (*m/z* 400–2000) were acquired in the Orbitrap with resolution R = 60000 with the number of accumulated ions being 1 × 10^6^. The 20 most intense ions were isolated and fragmented in the linear ion-trap (number of accumulated ions 1.5 × 10^4^) using collision induced dissociation. The lock mass option (polydimethylcyclosiloxane; *m/z* 445.120025) enabled accurate mass measurement in both the MS and MS/MS modes. In data-dependent LC-MS/MS experiments, dynamic exclusion was used with 60 s exclusion duration. MS conditions were 1.8 kV, capillary temperature of 250 °C, with no sheath and auxiliary gas flow. In the MS/MS mode, the ion selection threshold used was 500 counts and the activation Q-value was set at 0.25 and the activation time at 10 ms.

### Data analysis

MaxQuant 1.2.2.5 was used to identify proteins via automated identification of tandem mass spectra against the Beebase and Uniprot *Apis melifera* databases. Carbamidomethyl cysteine was set as fixed modification. Pyro-Gln, Pyro-Glu, oxidised methionine, N-acetylation and deamidation (NQ) were set as variable modifications. The precursor mass tolerance was set to 20 ppm, and fragment mass tolerance set to 0.8 Da. Two missed tryptic cleavages were allowed. Proteins were considered positively identified when they were identified with at least 1 tryptic peptide per protein and FDR of 0.01 (protein and peptide). Statistical analysis was performed using Perseus. Raw data were transformed (log 2) and imputated (width 0.3, Down shift 1.8) to replace missing values. Two-sample ANOVA was used to determine whether or not a protein significantly increased or decreased in abundance with at least 2-fold. The alpha level was set at 0.05. Global protein expression profiles were further analysed using the KEGG^42^ server.

## Additional Information

**How to cite this article**: Du Rand, E. E. *et al.* Detoxification mechanisms of honey bees (*Apis mellifera*) resulting in tolerance of dietary nicotine. *Sci. Rep.* **5**, 11779; doi: 10.1038/srep11779 (2015).

## Supplementary Material

Supplementary Tables 1, 2 and 3

## Figures and Tables

**Table 1:** Significantly up- or down-regulated metabolites in honey bees after three days of nicotine exposure.

Metabolite name	Fold change	p-value	Biological process
Nicotine	↑ 2.10	0.0024	Secondary metabolite
Cotinine	↑ 1.36	0.0296	Nicotine catabolite
Cotinine N-oxide	↑ 1.40	0.0305	Nicotine catabolite
1-palmitoylglycerophosphoinositol	↑ 1.79	0.0173	Lysolipid metabolism
4-hydroxyhippurate	↑ 5.40	0.0000	Benzoate metabolism
β-hydroxyisovalerate	↑ 1.33	0.0400	Val, Leu, Ile metabolism
Cytidine 5’-diphosphocholine	↓ 0.59	0.0072	Glycerolipid metabolism
Fumarate	↓ 0.72	0.0254	TCA cycle

(Welch’s two-sample Student t-Tests p-value < 0.05; False discovery rate q-value < 0.1).

**Table 2:** Summary of differentially expressed proteins in honey bees after three days of nicotine exposure. (ANOVA, p-value < 0.05).

Biological process/Function	Number of proteins Up Down	
Energy metabolism (oxidative phosphorylation)	15	4
Carbohydrate metabolism	16	2
Lipid metabolism	4	1
Amino acid metabolism	3	−
Glutathione metabolism	2	−
Detoxification and stress response	10	−
Transcription	1	−
Translation	7	4
Protein processing, modifications, folding and transport	6	5
Nucleotide metabolism	3	−
Muscle contraction, development	−	4
Nucleosome assembly	−	1
Cellular processes	3	4
Signal transduction	5	6
Cytoskeleton	−	3
Cofactor metabolism	−	1
Olfactory system	1	−
Unknown	20	24
TOTAL	96	59
