# Supplementary material for: Detoxification mechanisms of honey bees (Apis mellifera) resulting in tolerance of dietary nicotine
Source: Sci Rep. 2015 Jul 2;5:11779. doi: 10.1038/srep11779 (PMC4488760; doi:10.1038/srep11779)
Supplement: Supplementary Tables 1, 2 and 3 [file srep11779-s1.pdf]

Detoxification mechanisms of honey bees *(Apis mellifera)* resulting in tolerance of dietary nicotine

Esther E. du Rand, Salome Smit, Mervyn Beukes, Zeno Apostolides, Christian W.W. Pirk and Susan W. Nicolson

Supplementary Tables 1, 2 and 3.

Table 1 lists the up-regulated proteins with accession numbers, fold change and predicted functions.

Tables 2 lists the down-regulated proteins with accession numbers, fold change and predicted functions.

Table 3 lists the 12 cytochrome P450s identified in this study.

Supplementary Table 1. Significantly up-regulated proteins in honey bees after three days of nicotine exposure (ANOVA; $p \leq 0.05$; $q \leq 0.01$).

Beebase Identifier	Accession	Fold Change	p-value	Homologous Functions	Biological process/Function
**Energy metabolism**					
	NP_008084.1	1	0.0077	COX2_10414 cytochrome c oxidase subunit II	Oxidative phosphorylation
GB41139	XP_392983.1	2	0.0253	NADH dehydrogenase [ubiquinone] 1 alpha subcomplex subunit 8	Oxidative phosphorylation
GB41715	XP_625050.1	2	0.00698	cytochrome b-c1 complex subunit 2, mitochondrial-like	Oxidative phosphorylation
GB43629	XP_624801.1	2	0.0299	NADH dehydrogenase [ubiquinone] 1 beta subcomplex subunit 10-like	Oxidative phosphorylation
GB44693	XP_397201.2	2	0.0084	complement component 1 Q subcomponent-binding protein, mitochondrial-like	Oxidative phosphorylation
GB46369	XP_397047.2	2	0.0200	cytochrome c1, mitochondrial	Oxidative phosphorylation
GB46882	XP_394885.1	2	0.0033	NADH dehydrogenase [ubiquinone] iron-sulfur protein 3, mitochondrial	Oxidative phosphorylation
GB48163	XP_392437.2	2	0.0228	probable NADH dehydrogenase [ubiquinone] iron-sulfur protein 7, mitochondrial	Oxidative phosphorylation
GB49306	XP_625078.1	2	0.0042	ATP synthase subunit gamma, mitochondrial	Oxidative phosphorylation
GB50268	XP_623480.1	3	0.0141	ADH dehydrogenase [ubiquinone] iron-sulfur protein 8, mitochondrial isoform 2	Oxidative phosphorylation
GB52468	XP_001122467.1	2	0.0029	cytochrome b-c1 complex subunit 8-like	Oxidative phosphorylation
GB53010	XP_625254.1	2	0.0003	hypothetical protein LOC551541 cytochrome C activity predicted	Oxidative phosphorylation
GB55643	XP_392760.2	2	0.0215	ATP synthase subunit O, mitochondrial	Oxidative phosphorylation
GB55708	XP_624052.1	2	0.0477	NADH dehydrogenase [ubiquinone] 1 beta subcomplex subunit 8, mitochondrial-like	Oxidative phosphorylation
GB55782	XP_623441.1	2	0.0289	NADH dehydrogenase [ubiquinone] 1 alpha subcomplex subunit 6-like	Oxidative phosphorylation

Beebase Identifier	Accession	Fold Change	p-value	Homologous Functions	Biological process/Function
**Carbohydrate metabolism**					
GB42835	H9K434	1	0.0023	glycogen phosphorylase	Glycogenolysis
GB18109	XP_624353.1	2	0.0321	aldose/aldehyde reductase	Sucrose metabolism
	XP_003249233.1	3	0.0204	alpha,alpha-trehalose-phosphate synthase [UDP-forming] A isoform 1	Starch and sucrose metabolism
GB42385	XP_392401.1	3	0.0096	sorbitol dehydrogenase-like isoform 2	Sucrose metabolism
GB54661	XP_395366.2	2	0.0493	phosphoglucomutase	Sucrose metabolism
GB55537	XP_003251265.1	4	0.0149	transketolase	Pentose phosphate pathway
GB50272	XP_623497.1	2	0.0245	trans-1,2-dihydrobenzene-1,2-diol dehydrogenase-like isoform	Pentose & glucuronate interconversions
GB51283	XP_392104.4	4	0.0387	retinal dehydrogenase 1-like isoform 1	Pentose & glucuronate interconversions
GB15039	H9KD79	2	0.0071	enolase	Glycolysis
GB53566	XP_624025.3	3	0.0414	dihydrolipoyllysine-residue acetyltransferase component of PDHB	Glycolysis
GB55496	H9KJL4	3	0.0244	pyruvate dehydrogenase (PDHB)	Glycolysis
GB52073	XP_393545.2	2	0.0140	citrate synthase 1, mitochondrial-like	TCA cycle
GB19885	XP_003251170	2	0.0066	pyruvate carboxylase, mitochondrial-like	Glycolysis, TCA cycle, pyruvate metabolism
GB44430	XP_392679.4	2	0.0235	dihydrolipoyllysine-residue succinyltransferase component of 2-oxoglutarate dehydrogenase complex	TCA cycle
GB45258	H9KBQ4	2	0.0097	isocitrate dehydrogenase [NADP]	TCA cycle
	XP_624343.1	2	0.0256	succinyl-CoA ligase [ADP-forming] subunit beta, mitochondrial-like	TCA cycle
**Lipid metabolism**					
GB43706	XP_395130.4	2	0.0005	trans-2-enoyl-CoA reductase, mitochondrial-like	Fatty acid elongation
GB50970	XP_391843.1	2	0.0011	3-ketoacyl-CoA thiolase, mitochondrial-like	Fatty acid metabolism
	NP_001014994.1	3	0.0176	glycerol-3-phosphate dehydrogenase	Glycerophospholipid metabolism

Beebase Identifier	Accession	Fold Change	p-value	Homologous Functions	Biological process/Function
GB49999	XP_392060.2	4	0.0225	phosphatidylethanolamine-binding protein homolog F40A3.3-like isoform 1	
**Branched chain amino acid metabolism**					
GB55232	XP_001120471.1	2	0.0081	3-hydroxyacyl-CoA dehydrogenase type-2-like	Valine, leucine and isoleucine degradation
GB51283	XP_392104.4	4	0.0387	retinal dehydrogenase 1-like isoform 1	Valine, leucine and isoleucine degradation
GB50970	XP_391843.1	2	0.0011	3-ketoacyl-CoA thiolase, mitochondrial-like	Valine, leucine and isoleucine degradation
**Detoxification and Stress response**					
GB50265	NP_001171499.1	2	0.0265	glutathione S-transferase D1	Phase II conjugation with glutathione
GB40232	XP_003249289.1	4	0.0377	peroxiredoxin 1	Antioxidant activity
GB48634	XP_003250526.1	3	0.0460	phospholipid hydroperoxide glutathione peroxidase, mitochondrial isoform 2	Antioxidant activity
GB48905	NP_001153742.1	2	0.0003	glutathione S-transferase S1	Antioxidant activity; ROS conjugation with glutathione
GB49544	NP_001011578.1	3	0.0040	vitellogenin	Antioxidant activity
	XP_623090.2	2	0.0225	t-complex protein 1 subunit eta isoform 1	Chaperone proteins
	XP_394645.3	2	0.0009	hsc70-interacting protein 1-like isoform 1	Chaperone proteins
GB40976	NP_001153536.1	12	0.0021	heat shock protein 90	Chaperone proteins
GB42297	NP_001153520.1	2	0.0011	heat shock protein cognate 5	Belongs to the heat shock protein 70 family
GB47475	XP_395659.1	2	0.0241	protein lethal(2)essential for life-like isoform 1	Small heat shock protein (HSP20) family; stress response
**Glutathione metabolism**					
	XP_396817.3	2	0.0034	putative glutamate synthase [NADPH]-like isoform 1	
	XP_623576.3	2	0.0449	aminopeptidase N-like	
**Protein processing, modification, folding and transport**					
GB19208	XP_623373.2	3	0.0212	hypothetical protein LOC409613 isoform 2	Protein processing in ER
	XP_623831.2	3	0.0148	protein disulfide-isomerase	Protein processing in ER
	XP_393332.1	2	0.0216	nuclear protein localization protein 4 homolog	Protein processing in ER
	XP_397056.2	16	0.0301	ras-related protein Rab-14 isoform 1	Protein transport
	XP_003251420.1	5	0.0062	ras-related protein Rab-35-like	Protein transport
	XP_392689.2	2	0.0008	calreticulin isoform 1	Regulating calcium levels, protein folding & immune response

Beebase Identifier	Accession	Fold Change	p-value	Homologous Functions	Biological process/Function
**Nucleotide metabolism**					
	XP_003250779.1	2	0.0027	dihydropyrimidinase-like	Nucleotide metabolism
GB47503	XP_396399.3	2	0.0244	delta-1-pyrroline-5-carboxylate synthase-like	Nucleotide metabolism (polyamine synthesis from glutamate)
	XP_623921.1	1	0.0333	adenylate kinase 2, mitochondrial	Nucleotide metabolism
**Transcription and Translations**					
GB54355	XP_393344.3	2	0.0068	nuclease-sensitive element-binding protein 1 isoform X6	Regulation of transcription
	XP_392691.2	2	0.0173	longation factor 2-like isoform 1	Translation; Ribosomal translocation
	XP_393614.1	2	0.0027	60S ribosomal protein L7	Translation; Ribosome
	XP_623731.1	2	0.0283	40S ribosomal protein S3	Translation; Ribosome
	XP_393965.2	6	0.0030	40S ribosomal protein SA	Translation; Ribosome
	NP_001172073.1	2	0.0079	60S acidic ribosomal protein P1	Translation; Ribosome
	NP_001011628.1	2	0.0046	elongation factor 1-alpha	Translation; RNA transport
	XP_391880.2	2	0.0488	elongation factor Tu	Translation; Translation factor
**Olfactory system**					
	NP_001035313.1	3	0.0390	odorant binding protein 14	Olfactory system
**Cellular processes**					
	XP_003250979.1	10	0.0022	tubulin beta-3 chain-like	Cellular Processes; Transport and catabolism; Phagosome
	XP_623495.1	2	0.0048	v-type proton ATPase catalytic subunit A-like isoform 1	Cellular Processes; Transport and catabolism; Phagosome
	XP_392857.2	2	0.0140	lysosomal aspartic protease	Cellular Processes; Transport and catabolism; Lysosome
**Signal transduction**					
	XP_001120678.1	3	0.0075	toll-like receptor 13-like	Signalling
	XP_396167.1	2	0.0066	cAMP-dependent protein kinase type I regulatory subunit isoform 1	Signalling
	NP_001014430.1	2	0.0009	troponin C type I	Signal transduction; Calcium signaling pathway

Beebase Identifier	Accession	Fold Change	p-value	Homologous Functions	Biological process/Function
	XP_392097.4	3	0.0056	collagen alpha-1(IV) chain-like isoform 1	Signaling molecules and interaction; ECM-receptor interaction
	XP_003249128.1	2	0.0335	laminin subunit gamma-1	Signaling molecules and interaction; ECM-receptor interaction
**Unknown functions**					
	H9KCY6	1	0.0356	uncharacterized protein	
	XP_003251621.1	2	0.0140	uncharacterized protein CG7816-like	
	H9KLF2	1	0.0388	LOC409180	
	H9KMW0	1	0.0100	uncharacterized protein	
	XP_623625.1	2	0.0058	annexin-B9-like	
	XP_624913.1	2	0.0009	hypothetical protein LOC552534	
	XP_395866.2	2	0.0270	mitochondrial import inner membrane translocase subunit TIM44-like isoform 1	
	XP_623685.1	2	0.0484	hypothetical protein LOC408421	
	XP_396769.2	3	0.0066	chitinase-like protein Idgf4-like	
	XP_392501.2	2	0.0008	muscle-specific protein 20	
	XP_397374.4	2	0.0099	hypothetical protein LOC413936	
	XP_624452.3	2	0.0012	hypothetical protein LOC552071	
	XP_003249660.1	1	0.0126	hypothetical protein LOC100577682	
	XP_393212.3	4	0.0172	hypothetical protein LOC409713	
	XP_003250383.1	3	0.0157	uncharacterized protein C6orf168	
	XP_623682.3	2	0.0002	hypothetical protein LOC551211 isoform 2	
	XP_392328.4	2	0.0211	nidogen-2	
	XP_001120199.2	2	0.0283	hypothetical protein LOC725148	
	XP_001121552.2	2	0.0082	hypothetical protein LOC725740	
	XP_003251590.1	2	0.0081	hypothetical protein LOC100578389	

Supplementary Table 2. Significantly down-regulated proteins in honey bees after three days of nicotine exposure ($p \leq 0.05$; $q \leq 0.01$).

Beebase Identifier	Accession	Fold Change	p-value	Homologous Functions	Biological Process/Function
**Genetic information processing (Translation, transcription, protein processing and folding)**					
	XP_624359.3	2	0.0168	hypothetical protein LOC551973	Detects and degrades abnormal mRNA
	XP_001119835.1	2	0.0185	dnaJ homolog subfamily A member 1	Chaperone protein
	XP_623090.2	2	0.0078	t-complex protein 1 subunit eta isoform 1	Chaperone protein
	XP_623603.1	2	0.0097	neutral alpha-glucosidase AB-like isoform 2	Protein processing in ER
	XP_623373.2	3	0.0127	hypothetical protein LOC409613	Protein processing in ER
	XP_392689.2	2	0.0132	calreticulin isoform 1	Protein processing in ER
	XP_395299.2	2	0.0391	translationally-controlled tumor protein homolog isoform 1	Translation
	XP_624911.2	2	0.0171	probable leucyl-tRNA synthetase, mitochondrial-like isoform 2	Translation; Aminoacyl-tRNA biosynthesis
	XP_624943.2	3	0.0092	40S ribosomal protein S7	Translation; Ribosome
**Signal transduction**					
	XP_003250350.1	2	0.0375	plasma membrane calcium-transporting ATPase 3 pmca	Signal transduction
	NP_001010975.1	2	0.0022	ADP/ATP translocase	Signal transduction; Calcium signalling pathway
	XP_393851.3	4	0.0013	calcium-transporting ATPase sarcoplasmic/ER type isoform 1	Signal transduction; Calcium signalling pathway
	XP_396118.4	4	0.0318	laminin subunit alpha, partial	Signalling molecules and interaction; ECM-receptor interaction
	XP_393220.4	2	0.0066	basement membrane-specific heparan sulfate proteoglycan core protein	Signalling molecules and interaction; ECM-receptor interaction
	XP_396960.2	3	0.0339	phosphate carrier protein, mitochondrial-like isoform 1	Signal transduction
**Muscle contraction, development**					
	XP_392125.2	5	0.0462	hypothetical protein LOC408583 isoform 1	Muscle contraction
	NP_001035346.1	1	0.0428	troponin I	Muscle contraction
	XP_394758.3	2	0.0096	muscle LIM protein Mlp84B-like isoform 1	Muscle development
	XP_396670.3	2	0.0249	lamin Dm0-like	Myosin tail

Beebase Identifier	Accession	Fold Change	p-value	Homologous Functions	Biological Process/Function
**Other proteins**					
	XP_624807.2	2	0.0088	neurochondrin homolog	Carbohydrate metabolism
	NP_001171540.1	3	0.0143	catalase	Cellular Processes; Transport and catabolism; Peroxisome
	XP_623357.1	4	0.0218	transketolase isoform 2	Carbohydrate metabolism; Pentose phosphate pathway
	XP_391944.4	6	0.0033	talin-1-like	Cellular Processes; Cell communication; Focal adhesion
	XP_003251676.1	3	0.0008	v-type proton ATPase subunit H isoform 2	Cellular Processes; Transport and catabolism; Phagosome
	XP_393413.2	2	0.0339	ferrochelatase, mitochondrial isoform 1	Cofactor and vitamin metabolism
	XP_396777.4	2	0.0320	spectrin beta chain	Cytoskeleton
	XP_396653.1	2	0.0221	tropomyosin-2-like	Cytoskeleton
	XP_391961.2	2	0.0022	tropomyosin-1-like	Cytoskeleton
	XP_395789.1	2	0.0268	NADH dehydrogenase [ubiquinone] iron-sulfur protein 6, mitochondrial	Oxidative phosphorylation
	XP_392983.1	2	0.0423	NADH dehydrogenase [ubiquinone] 1 alpha subcomplex subunit 8	Oxidative phosphorylation
	NP_001170961.1	2	0.0267	cytochrome c	Oxidative phosphorylation
	NP_008084.1	1	0.0298	COX2_10414 cytochrome c oxidase subunit II	Oxidative phosphorylation
	XP_624898.3	2	0.0031	protein KIAA0664 homolog	Involved in proper cytoplasmic distribution of mitochondria
	NP_001011614.1	3	0.0012	phospholipase A2 precursor	Lipid metabolism
	XP_003251221.1	7	0.0045	histone H4-like	Nucleosome assembly
**Other proteins**					
	XP_394370.1	2	0.0239	chymotrypsin-1	Peptidase; unknown
	XP_001123295.2	4	0.0138	trypsin-1-like, partial	Peptidase; unknown
	NP_001078815.1	2	0.0186	apidermin 2	Transporter ; unknown
	H9K7Z1	2	0.0087	uncharacterized protein	Unknown
	NP_001165860.1	2	0.0076	cuticular protein analogous to peritrophins 3-C	Unknown
	XP_397188.3	2	0.0060	antithrombin-III serpin-5	Unknown
	XP_003250139.1	1	0.0004	hypothetical protein LOC100577527	Unknown
	XP_003250642.1	2	0.0082	sarcalumenin-like	Unknown

Beebase Identifier	Accession	Fold Change	p-value	Homologous Functions	Biological Process/Function
	XP_001120827.2	2	5.34E-05	importin-5-like	Unknown
	XP_395618.4	2	0.0011	oligopeptidase A-like	Unknown
	XP_392962.1	2	0.0480	receptor of activated protein kinase C 1 isoform 1	Unknown
	NP_001011640.1	1	0.0067	take-out-like carrier protein	Unknown
	XP_625294.1	2	0.0358	hypothetical protein LOC552685	Unknown
	XP_395236.2	2	0.0415	kinesin heavy chain isoform 1	Unknown
	XP_393632.2	6	0.0292	plasma glutamate carboxypeptidase-like isoform 1	Unknown
	H9JZK4	2	0.0177	uncharacterized protein	Unknown
	HXP_392617.4	2	0.0098	neurotrimin	Unknown
	XP_392361.4	2	0.0284	hypothetical protein LOC408830 isoform 1	Unknown
	H9KC31	3	0.0330	uncharacterized protein	Unknown
	XP_001120418.1	3	0.0010	hypothetical protein LOC725454	Unknown
	H9KFR6	1	0.0150	uncharacterized protein	Unknown
	H9KJ51	1	0.0067	uncharacterized protein	Unknown
	H9KKA0	1	0.0115	uncharacterized protein	Unknown
	XP_623146.2	2	0.0344	hypothetical protein LOC412543 isoform 1	Unknown

Supplementary Table 3. Cytochrome P450 enzymes identified in honey bees after three days of nicotine exposure.

Enzyme Identified	Specific Functions
CYP18A1	Unknown
CYP336A1	Unknown
CYP4G11	Protection against oxidative stress
CYP6AS10	Detoxification; Quercetin metabolizing; constitutively expressed at high levels[1,2]
CYP6AS13	Detoxification; Quercetin metabolizing[1]
CYP6AS15	Detoxification; Quercetin metabolizing; constitutively expressed at high levels[1,2]
CYP6AS3	Detoxification; Quercetin metabolizing[1]
CYP6BD1	Detoxification[3]
CYP9Q1	Detoxification; Pyrethroid and organophosphate metabolizing[4]
CYP9Q2	Detoxification; Pyrethroid and organophosphate metabolizing; constitutively expressed at low levels [4]
CYP9Q3	Detoxification; Pyrethroid and organophosphate metabolizing; constitutively expressed at low levels[4]
CYP9R1	Unknown

References for Supplementary Table 3

1. Mao, W. *et al.* Quercetin-metabolizing CYP6AS enzymes of the pollinator *Apis mellifera* (Hymenoptera: Apidae). *Comp. Biochem. Physiol. B Biochem. Mol. Biol.* **154,** 427–434 (2009).
2. Johnson, R. M. *et al.* Ecologically appropriate xenobiotics induce cytochrome P450s in *Apis mellifera*. *PLoS ONE* **7,** e31051 (2012).
3. Mao, W., Schuler, M. A. & Berenbaum, M. R. Honey constituents up-regulate detoxification and immunity genes in the western honey bee Apis mellifera. *Proc. Natl. Acad. Sci.* **110,** 8842–8846 (2013).
4. Mao, W., Schuler, M. A. & Berenbaum, M. R. CYP9Q-mediated detoxification of acaricides in the honey bee (Apis mellifera). *Proc. Natl. Acad. Sci.* **108,** 12657–12662 (2011).
